# Type I interferonopathies in pediatric rheumatology

**DOI:** 10.1186/s12969-016-0094-4

**Published:** 2016-06-04

**Authors:** Stefano Volpi, Paolo Picco, Roberta Caorsi, Fabio Candotti, Marco Gattorno

**Affiliations:** U.O. Pediatria 2, Istituto Giannina Gaslini, Genoa, Italy; Division of Immunology and Allergy, University Hospital of Lausanne, Lausanne, Switzerland

**Keywords:** Type I interferon, Type I interferonopathies, Familial lupus, SAVI, CANDLE, Aicardi-Goutières syndrome

## Abstract

Defective regulation of type I interferon response is associated with severe inflammatory phenotypes and autoimmunity. Type I interferonopathies are a clinically heterogenic group of Mendelian diseases with a constitutive activation of this pathway that might present as atypical, severe, early onset rheumatic diseases. Skin vasculopathy with chilblains and livedo reticularis, interstitial lung disease, and panniculitis are common. Recent studies have implicated abnormal responses to nucleic acid stimuli or defective regulation of downstream effector molecules in disease pathogenesis. As observed for IL1-β and autoinflammatory diseases, knowledge of the defects responsible for type I interferonopathies will likely promote the development of targeted therapy.

Nature is nowhere accustomed more openly to display her secret mysteries than in cases where she shows traces of her workings apart from the beaten path: nor is there any better way to advance the proper practice of medicine than to give our minds to the discovery of the usual law of Nature by careful investigation of cases of rarer forms of disease. For it has been found, in almost all things, that what they contain of useful or applicable nature is hardly perceived unless we are deprived of them, or they become deranged in some way

-William Harvey (1651)

## Background

In recent years it has been increasingly recognised that patients presenting early in infancy with persistent or recurrent inflammatory phenotypes might suffer from underlying genetic conditions. Systemic autoinflammatory diseases (SAIDs) such as cryopyrin-associated periodic syndrome (CAPS), tumor necrosis factor (TNF) receptor-associated periodic syndrome (TRAPS) and familial Mediterranean fever (FMF) are examples of such entities. Moreover, it is common for practicing pediatric rheumatologists to observe patients who only partially fit classic diagnostic criteria for known, well-defined clinical conditions or who present atypical characteristics in term of severity, disease onset and treatment response, and thus represent both diagnostic and therapeutic challenges.

Today, the differential diagnosis of such clinical cases has to include a recent new class of mendelian inherited disorders linked to defective regulation of type I interferons (IFN), named type I interferonopathies [1]. These conditions initially included i) Aicardi-Goutières syndrome (AGS), ii) familial chilblain lupus, iii) spondyloenchondrodysplasia (SPENCD) and iv) monogenic forms of systemic lupus erythematosus (SLE). An increasing number of genetic diseases belonging to this family have later been discovered, including the Proteasome Associated Autoinflammatory Syndromes (PRAAS), IFN-stimulated gene 15 (*ISG15*) deficiency, Singleton-Merten syndrome and its atypical presentation (SMS), and stimulator of IFN genes (STING)-associated vasculopathy with onset in infancy (SAVI).

The objective of this review is to summarize the clinical and molecular features of type I interferonopathies with a special focus on the ones more likely to be encountered by pediatric rheumatologists.

### Type I IFN pathway activation and signalling

IFNs are secreted molecules that represent one of the cell’s first lines of defense against pathogens. Their existence, and the same name interferon, was first proposed by Isaacs and Lindenmann more than 50 years ago [2], following the observation that the supernatant of cells incubated with heat-inactivated influenza virus was able to “interfere” with viral infections if added to another cell culture. In the following years the understanding of IFNs effector mechanism shed the light on a highly conserved antiviral response required for the survival of the host.

**Fig. 1:**
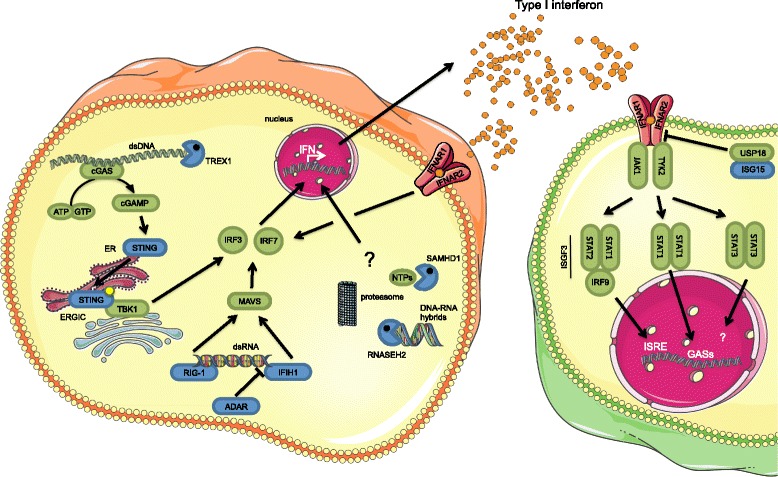
Cytoplasmic nucleic acid recognition and type I IFN pathway activation. Scheme of cytoplasmic nucleotide sensing, type I IFN secretion and autocrine and paracrine IFNAR activation. Colored in blue are some of the proteins mutated in type I interferonopathies. Pathways currently not fully understood are identified with a question mark. cGAMP: cyclic di-GMP-AMP, cGAS: cyclic GMP-AMP synthase, ER: endothelial reticulum, ERGIC: endothelial reticulum-Golgi intermediate compartment, IFIH1: IFN-induced helicase C domain-containing protein 1 (also known as MDA5), IFNAR: interferon-α receptor, ISG15: interferon-stimulated gene 15, MAVS: mitochondrial antiviral-signaling protein, RIG-I: retinoic acid-inducible gene 1, SAMHD1: deoxynucleoside triphosphate triphosphohydrolase SAM domain and HD domain 1, STING: stimulator of interferon genes, TBK1: TANK-binding kinase 1, TREX1: DNA 3ʹ repair exonuclease 1, USP18: ubiquitin-specific protease 18

Viral and bacterial pathogens that induce a type I IFN response are sensed in the cytoplasm or endosomes of infected cells by different pattern recognition receptors, which include Toll-like receptors (TLRs), RIG-I-like receptors (RLRs), NOD-like receptors (NLRs) and a growing family of cytoplasmic DNA receptors such as AIM2, cyclic GMP-AMP synthase (cGAS) and γ-IFN-inducible protein 16 (IFI16) [3, 4]. The role of cytoplasmic nucleic acid sensors has become increasingly evident in the pathogenesis of type I interferonopathies. In particular, cytoplasmic dsDNA has been shown to interact with the enzyme cGAS, which catalyzes the production of the non-canonical cyclic dinucleotide di-GMP-AMP (cGAMP) [5]. cGAMP binds and activates the STING protein, which, following activation, translocates from the endothelial reticulum (ER) to the ER-Golgi intermediate compartments (ERGIC) [6] where the signal is propagated through the phosphorylation of the TANK-binding kinase 1 (TBK1) and of a family of protein called IFN regulatory factors (IRF), in particular IRF3 [7], that translocate to the nucleus and induce the transcription of IFN-β [8] and IRF7, which is responsible for IFN-α induction and autocrine type I IFN signalling amplification [9] (Fig. 1). Excessive activation of the cellular nucleotides sensor system, therefore, can results in increase production of IFN and inappropriate inflammation.

Type I IFNs are represented by 13 IFN-α with very similar and highly conserved sequences of 161–167 aa [10] and a single IFN-β.

Two different main functions of type I IFN pathway are described: the antiviral activity and the antiproliferative activity. While the antiviral activity is accomplished by all IFNs even at a very low concentration and occurs in most cells, the antiproliferative activity is highly cell-type specific and is a function of the levels of expression of the IFN and its cellular receptors, as well as of the receptor binding affinity of IFN.

Not surprisingly, given the conservation of type I IFN pathway across species, germline mutations that impair such functions are linked to genetic susceptibility to severe viral diseases, such as herpes virus encephalitis in patients with mutations of *UNC93B*, *TLR3*, *TRAF3*, *TRIF* and *TBK1*, or life-threatening influenza in patients with mutations in *IRF7* [11–13].

Type I IFNs bind to the same heterodimeric receptor that is expressed by all nucleated cells and is constituted by the subunits IFN-α receptor 1 (IFNAR1) and IFNAR2. Binding of the IFN to one receptor subunit induces dimerization of IFNAR1 and IFNAR2, phosphorylation of the Janus Kinases (JAK), TYK2 and JAK1, and activation of different STAT family members (Fig. 1).

As mentioned above, the different effector functions of type I IFN depend on i) the different affinities of the ligand to the receptor subunits [14–16]; ii) receptor expression by target cells; iii) IFN expression by the tissue. Thus the biological activity of IFN response is tightly regulated despite the existence of a single receptor.

### Type I IFN dysregulation

In the 1970s Gresser and colleagues [17] were the firsts to suggested the existence of possible pathogenic effects of IFN: newborn animals injected with high doses of IFN presented the same severe growth retardation, liver lesions, glomerulonephritis and mortality of animals infected by lymphocytic choriomeningitis virus (LCMV) suggesting that IFN itself was responsible for the induction of those lesions. Moreover, the Authors showed how anti-IFN antibody therapy could prevent the development of glomerulonephritis in mice infected with LCMV [18].

Most of the genes that have been shown to be mutated in type I interferonopathies are involved in the metabolism of nucleic acids or their recognition machinery, i.e. the receptors that are responsible for sensing pathogen-derived nucleic acids and the related downstream mediators (Table 1). In particular, mutations that inhibit the function of nucleic acid-related enzymes are responsible for AGS and the damaged players include: DNA 3ʹ-repair exonuclease 1 (TREX1) and Ribonuclease H2 (RNASE H2) complex, both nucleases that degrade DNA and DNA-RNA hybrid molecules preventing the accumulation of endogenous nucleic acids in the cytoplasm [19–21], SAMHD1, a protein that restricts the availability of cytosolic deoxynucleotides (dNTPs) [22, 23] and adenosine deaminase acting on RNA 1 (ADAR1), an enzyme that edits endogenous dsRNA preventing its recognition by the cytosolic receptor IFIH1 [24, 25]. Similarly, activating mutations of nucleic acid receptors IFIH1 [26–28] and RIG-I [29] cause autosomal dominant AGS and Singleton-Merten syndrome interferonopathies, while activating mutations of STING cause SAVI syndrome in the absence of chronic infectious triggers [30, 31].

**Table 1:** Type I interferonopathies. Mutated gene, protein function, pattern of inheritance and main symptoms of know type I interferonopathies

Disease	Gene	Protein function	Inheritance	Symptoms
Aicardi-Goutières syndrome (AGS)1	*TREX*-*1*	3′-5′ DNA exonuclease	AR and AD	Classical AGS
AGS2	*RNASEH2B*	Components of Rnase H2 complex. Removes ribonucleotides from RNA-DNA hybrids	AR	Classical AGS
AGS3	*RNASEH2C*			Classical AGS
AGS4	*RNASEH2A*			Classical AGS with dysmorphic features
AGS5	*SAMHD1*	Restricts the availability of cytosolic deoxynucleotides	AR	Mild AGS, mouth ulcer, deforming arthropathy, cerebral vasculopathy with early onset stroke
AGS6	*ADAR*	Deaminates adenosine to inosine in endogenous dsRNA preventing recognition by MDA5 receptor	AR and AD	Classical AGS, bilateral striatal necrosis
AGS7	*IFIH1*	Cytosolic receptor for dsRNA	AD	Classical or mild AGS, asymptomatic
Retinal vasculopathy with cerebral leukodystrophy (RVCL)	*TREX*-*1*	3′-5′ DNA exonuclease	AD	Adult-onset loss of vision, stroke, motor impairment, cognitive decline, Raynaud and liver involvement
Spondyloenchondrodysplasia (SPENCD)	*ACP5*	Lysosomal phosphatase activity	AR	Spondyloenchondrodysplasia, immune disregulation and in some cases combined immunodeficiency
STING associated vasculopathy with onset in infancy (SAVI)	*TMEM173*	Transduction of cytoplasmic DNA-induced signal	AD	Systemic inflammation, cutanous vasculopathy, pulmonary inflammation
Proteasome Associated Autoinflammatory Syndromes (PRAAS)	*PSMB8*	Part of the proteasome complex	AR	Autoinflammation, lipodistrophy, dermatosis, hyper-immunoglobulinemia, joint contractures (JMP), short stature
ISG15 deficieny	*ISG15*	Stabilizes USP18, a negative regulator of type I interferon	AR	Brain calcifications, seizures, mycobacterial susceptibility
Singleton-Merten syndrome (SMS)	*IFIH1*	Cytosolic receptor for dsRNA	AD	Dental dysplasia, aortic calcifications, skeletal abnormalities, glaucoma, psoriasis
Atypical SMS	*DDX58*	Cytosolic receptor for dsRNA	AD	Aortic calcifications, skeletal abnormalities, glaucoma, psoriasis
Trichohepatoenteric syndrome (THES)	*SKIV2L*	RNA helicase	AR	Severe intractable diarrhea, hair abnormalities (trichorrhexis nodosa), facial dysmorphism, immunodeficiency in most cases

*ADAR1* adenosine deaminase acting on RNA 1, *ACP5* Acid Phosphatase 5, Tartrate Resistant, *AGS* Aicardi-Goutières syndrome, *DDX58* DEAD Box Protein 58, *IFIH1* IFN-induced helicase C domain-containing protein 1 (also known as MDA5), *ISG15* Interferon-stimulated gene 15, *PSMB8* Proteasome subunit beta type-8, *RNASEH2* Ribonuclease H2, *RVCL* Retinal vasculopathy with cerebral leukodystrophy, *SAMHD1* deoxynucleoside triphosphate triphosphohydrolase SAM domain and HD domain 1, *SPENCD* spondyloenchondrodysplasia, *SAVI* STING associated vasculopathy with onset in infancy, *PRAAS* Proteasome Associated Autoinflammatory Syndromes, *SMS* Singleton-Merten syndrome, *THES* Trichohepatoenteric syndrome, *TMEM173* transmembrane Protein 173, *TREX1* DNA 3ʹ - repair exonuclease 1

These findings strongly support a model where the activation of type I IFN pathway is caused by either an increase in the burden of nucleic acids derived from endogenous retroelements or by the constitutive activation of nucleic acid receptors and mediators [32]. A different mechanism is involved in the case of *ISG15* deficiency: type I IFN is tightly regulated by suppressive signals in order to prevent toxicity driven by downstream effector functions such as the ubiquitin-specific protease 18 (USP18). A defect in USP18-mediated attenuation of type I IFN response has been shown in patients with *ISG15* deficiency, a disease characterized by intracranial calcifications, seizures, atypical mycobacteria infection susceptibility, autoantibodies and increased IFN-α or increased expression of IFN stimulated genes in peripheral blood, a biomarker known as type I IFN signature, detected by standard real-time PCR or micro-array technique [33].

### Clinical features and molecular defects

#### Familial systemic lupus erithematosus

Rare cases of monogenic form of SLE (OMIM 152700) have been reported in patients harboring mutations in *TREX1* (autosomal dominant (AD)), *SAMHD1* (AD), *ACP5* (autosomal recessive (AR), discussed later), *DNase1* (AD), *DNase1L3* (AR), protein kinase C δ (*PRKCD*) (AR) and complement deficiency of C1q/r/s, C4 subunits (AR). A minority of patients with C2 and C3 deficiency (around 10 %) may develop a less severe form of lupus-like disease [34] (Table 2). With the exception of *DNase1*, *DNase1L3*, *PRKCD* deficiencies and complement deficiencies (for which no information on IFN expression is available), an increase in type I IFN activity was documented in the most part of affected patients.

**Table 2:** Monogenic forms of SLE

Disease	Gene		Protein function	Inheritance	Clinical presentation
Monogenic SLE	*TREX1*		3′-5′ DNA exonuclease	AD (AR in few cases)	SLE
	*C1q*	*C1qA*	Central pattern-recognition molecule in the classical pathway of the complement system	AR	SLE, membranous proliferative GN, arthritis, bacterial infections
		*C1qB*			
		*C1qC*			
	*C1r*		Components of the C1 complex in the classical pathway of the complement system	AR	SLE, RA-like arthritis, sinopulmunary infections
	*C1s*				SLE, Hashimoto’s thyroiditis, autoimmune hepatitis
	*C2*		Component of the classical pathway of the complement system	AR	SLE in a minority of affected individual. Arthritis, malar rash, discoid rash.
	*C3*		Major complement component, involved in all three pathways of activation	AR	Upper and lower respiratory tract infection, SLE in a minority of affected individual.
	*C4A*		Component of the classical pathway of the complement system	AR	SLE, type 1 diabetes mellitus, glomerulonephritis
	*Dnase1*		Endonuclease present in tissues, serum and body fluids	AD	SLE, Sjögren syndrome, antinucleosomal autoantibodies
	*DNase1L3*		Endonuclease, homologue to *Dnase1*	AR	Pediatric onset SLE, lupus nephritis, hypocomplementemic urticarial vasculitis syndrome HUVS.
	*ACP5*		Lysosomal phosphatase activity	AR	Skeletal dysplasia (SPENCD), SLE, Sjögren syndrome, Raynaud
	*PRKCD*		Serine/threonine kinase implicated in the control of cell proliferation and apoptosis	AR	Pediatric onset SLE, lupus nephritis
	*IFIH1*		Cytosolic receptor for dsRNA	AD	SLE with IgA deficiency, mild lower limb spasticity
Chilblain lupus	*TREX*-*1*		3′-5′ DNA exonuclease	AD	Chilblain lesions, skin ulcers, loss of ear cartilage
	*SAMHD1*		Restricts the availability of cytosolic deoxynucleotides	AR and AD	Chilblain lesions, photosensitivity

AD autosomal dominant, AR autosomal recessive, GN glomerulonephritis, *ACP5* Acid Phosphatase 5, Tartrate Resistant, HUVS Hypocomplementemic urticarial vasculitis syndrome, *IFIH1* IFN-induced helicase C domain-containing protein 1 (also known as MDA5), *PRKCD* Protein Kinase C Delta, *SAMHD1* deoxynucleoside triphosphate triphosphohydrolase SAM domain and HD domain 1, *TREX1* DNA 3ʹ repair exonuclease 1

SLE is known to be associated with an increase in plasma type I IFN levels since at least the early eighties [35–37]. The activation of type I IFN pathway has been shown to correlate with disease activity [38] and some increased IFN-α activity has been found also in family members of SLE patients [39]. Further evidences towards a causal role of type I IFN in at least some of the clinical presentations of SLE came from the observation that patients treated with recombinant human IFN-α for malignancies or viral hepatitis can develop SLE symptomatology that usually resolves with the discontinuation of the drug [40, 41]. Interestingly, TNF has been shown to have an inhibitory effect on IFN-α induction in peripheral blood mononuclear cells derived from both healthy controls and SLE patients [42]. Furthermore, treatment with anti-TNF therapies induces the transcription of type I IFN-stimulated genes in vivo. Consistent with these findings is the rare observation of SLE development in patients treated with anti-TNF therapies. This can be explained either by an “unmasking” effect in predisposed patients, or a drug-induced effect, a clinical entity referred as *anti*-*TNF induced lupus*, ATIL [43].

AD defects in the nuclease TREX1 represent the most common cause of monogenic lupus with a frequency of 0.2–2 % in the adult SLE population [44–46] and have been linked to a particular form of SLE presenting with skin lesions of the extremities induced by cold exposure, called chilblains (CHBL1, OMIM610448) [47–49]. Familial SLE cases due to AR homozygous mutations of TREX1 have been also reported [46].

Of note, AD frameshift mutations in the C-terminal portion of TREX1 have been shown to result also in the retinal vasculopathy with cerebral leukodystrophy (RVCL; OMIM 192315), a syndrome characterized by loss of vision, stroke, dementia and in some cases glomerulopathy and Raynaud’s disease [50]. An increased type I IFN signature has been described in the peripheral blood of such patients [51].

Mutations in *SAMHD1* have also been reported in a few families affected by chilblain lupus with and without central nervous system involvement (CHBL2, OMIM 614415) [52, 53]. Arthritis, mental retardation and microcephaly have also been observed in patients with mutations in *SAMHD1*.

AR deletions of one bp in the *DNase1L3* gene leading to loss of RNA transcripts have been described in 17 cases of juvenile onset SLE from 6 different families from Saudi Arabia (OMIM 614420). About 65 % of affected patients presented with positive ANAs, high frequency of ANCAs and lupus nephritis [54]. Complete loss of nuclease activity was documented in mutant proteins. Homozygous loss-of-function mutations of *DNase1L3* have been described also in five patients from two families who were diagnosed with severe hypocomplementemic urticarial vasculitis syndrome (HUVS) and presenting clinically with recurrent urticaria, fatigue, fever, continuous acute phase reactant elevation and kidney involvement (mostly lupus nephritis class II or III) [55]. In our center we followed one case with early onset recurrent fever, urticarial vasculitis-like skin lesions, necrotizing ANCA-associated glomerulonephritis, enlarged lymphnodes, chronic anemia, articular effusion and chilblains (manuscript in preparation).

Finally, loss of function heterozygous mutations of the nuclease *DNase1* have been reported in two children with early onset SLE, and high titer anti-nucleosomal and anti-dsDNA autoantibodies. Subclinical Sjögren syndrome and IgG mesangial deposition at kidney biopsy were present in one case. The enzymatic activity of the mutant protein was low compared to controls [56].

Primary complement defects are associated with an increased risk of developing SLE estimated between 93 % of cases for C1q deficiency (OMIM 613652), 75 % for C4A deficiency (OMIM 614380) and 66 % for C1r and C1s (OMIM 216950) [57]. The pattern of inheritance is AR and kidney (membranous proliferative glomerulonephritis) as well as skin involvement are common [58], together with an increased susceptibility for pyogenic infections. The main mechanisms of the disease is thought to be linked to a defective immune complex processing and clearance [59], which results in activation of autoreactive B cells [60] leading to a decreased tolerance [61], together with a failure to control INF-α production by plasmacytoid dendritic cells [62].

#### Sting associated vasculopathy with onset in infancy

SAVI (OMIM 615934) is a type I interferonopathy caused by sporadic or familial autosomal heterozygous mutations of the Transmembrane Protein 173 (*TMEM173*) gene. After its first recent characterization [30], several new cases have been reported thus suggesting that the disease incidence may not be extremely uncommon [31, 63–65]. SAVI is clinically characterized by systemic features (e.g. fever spikes, malaise, chronic anemia, growth failure), in addition to cutaneous involvement and interstitial lung disease [30, 31, 63–65].

**Fig. 2:**
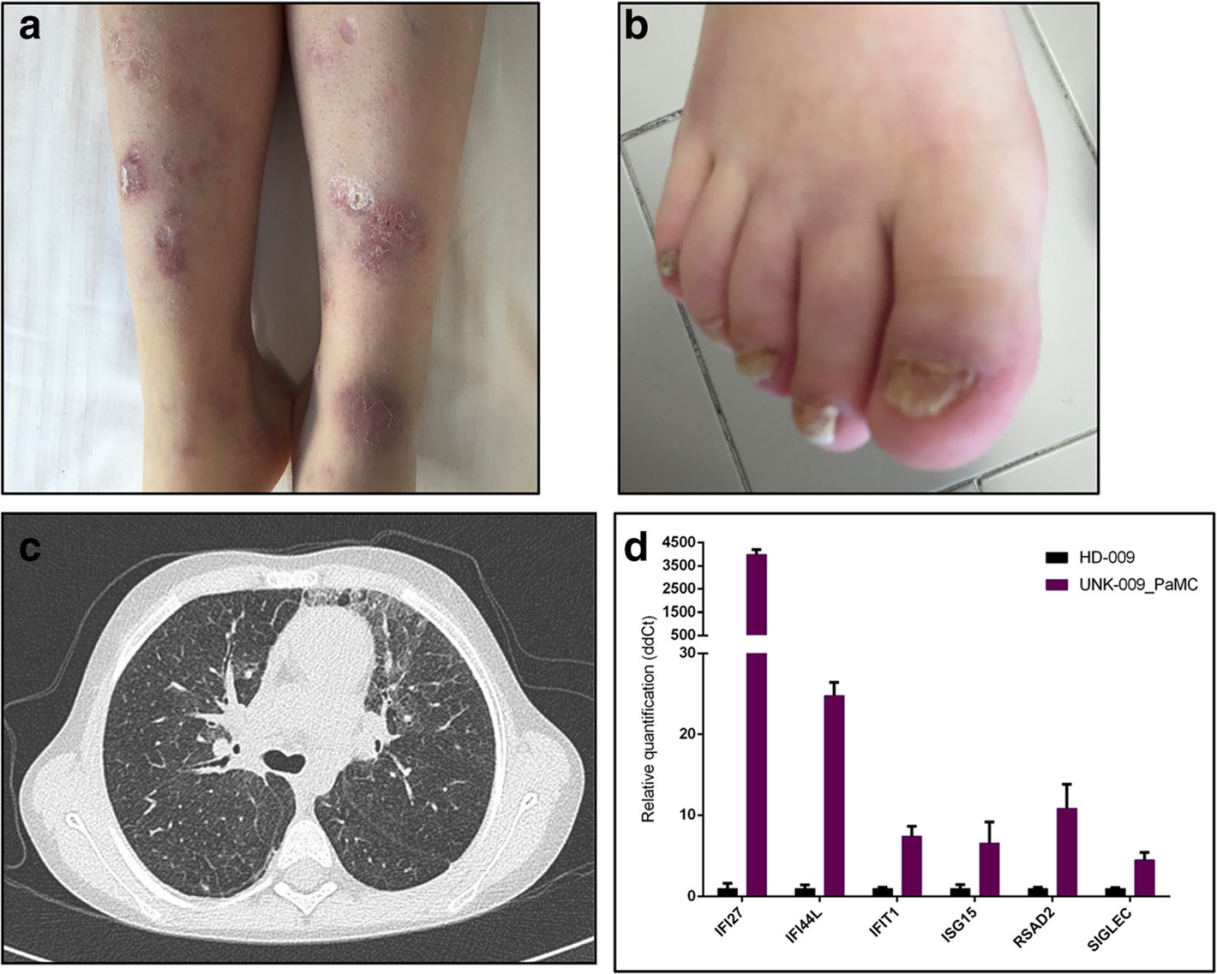
Clinical presentation and blood interferon signature of a SAVI patient. Purpuric plaques with ulcerative evolution (panel **a**), onychodystrophy (panel **b**), CT scan revealing focal thickening of the interlobular septa with areas of ground glass opacities (panel **c**), and peripheral blood type I interferon signature (panel **d**) (assessed as described [67]) in a patient with SAVI syndrome

Skin lesions are characterized by an early onset. They are usually localized at the face with a papulo-follicular appearance and at acral zones (fingers, ears, tip of the nose) where they may consist of erythematous or purpuric plaques and nodules, livedo reticularis, painful ulcerative lesions evolving onto eschars with tissue loss or digital amputation (Fig. 2, panel a and b). Raynaud phenomenon has been also reported: at capillaroscopic examination, nailfold capillary tortuosity may be observed, albeit without a clear scleroderma pattern. Periungual erythema and onychodystrophy are commonly observed and may be a heralding symptom of the disease [30, 63, 65]. Notably cold exposure may trigger cutaneous flares.

Histopathologic analysis of skin biopsy specimens is consistent with diffuse capillary wall inflammation with neutrophilic infiltrates and microthrombotic changes. No signs of vasculitis or granulomatosis have been reported. Mucosal lesions, such as oral ulcers, aphthosis and nasal septum perforation may be present.

Pulmonary involvement is not overtly symptomatic in the early phases of the disease; it consists of interstitial lung disease leading to lung fibrosis [30, 31] (Fig. 2, panel c). Cough and tachypnea are commonly reported. Notably, in one case observed at our Center, a concomitant viral pneumonia triggered a life-threatening acute respiratory failure strongly mimicking lymphocytic interstitial pneumonia (LIP). Chest X-ray usually shows lung hyperinflation. Computed tomography, the gold-standard diagnostic tool of the interstitial lung disease [66] will show a wide spectrum of lesions (septal thickening, ground-glass opacifications, bronchiectasias, etc.). Hilar and paratracheal lymphadenopathy is often associated (Fig. 2). Lung-biopsy specimens show scattered mixed lymphocytic inflammatory infiltrate.

Low-titer autoantibodies (e.g. antinuclear antibody, anticardiolipin antibodies and antibodies against β2 glycoprotein I) are found; notably, the presence of antineutrophils cytoplasmic antibodies (cANCA) associated with SAVI clinical features may lead to misdiagnosis of childhood granulomatosis with polyangiitis [65].

So far, peripheral blood type I interferon signature represents the most useful diagnostic tool to suspect SAVI syndrome, which requires molecular analysis for confirmation (Fig. 2, panel d) [67]. As already discussed, SAVI syndrome is due to gain of function mutation of the STING protein, which is involved in signal transmission from the cGAS DNA receptor. The mechanism underling the constitutive activation of STING seems to be a deregulated trafficking from the ER to the ERGIC independently of cGAMP binding, leading to an increased and chronic hyper secretion of IFN-β (Fig. 1) [6].

#### Proteasome-associated autoinflammatory syndromes

PRAAS (OMIM 256040) are a group of distinct clinical entities that have recently been recognised to share a common molecular cause. They include joint contractures, muscle atrophy, microcytic anemia and panniculitis-induced lipodystrophy syndrome (JMP), Nakajo-Nishimura syndrome (NNS, also referred to as Japanese autoinflammatory syndrome with lipodystrophy, JASL) and chronic atypical neutrophilic dermatosis with lipodystrophy and elevated temperature syndrome (CANDLE).

All these syndromes are characterized by the early onset of nodular, pernio-like, violaceous skin lesions with atypical neutrophil infiltrates, muscle atrophy, lipodystrophy, failure to thrive and deformities of the hands and feet due to joint contractures. Recurrent periodic fever episodes and elevated-acute phase reactant levels are usually present. Other common features are hepatosplenomegaly, prominent abdomen, basal ganglia calcifications, hypochromic anemia, increased IgG, absence or in few cases intermittent-low titer autoantibodies. Acanthosis nigricans and hypertriglyceridemia have been also reported [68–71].

The original form of PRAAS was described in the Japanese population by Nakajo with features of secondary hypertrophic osteoperiostosis with pernio [72]. It was later recognised that lipodystrophy and inflammation were a prominent feature [73–75]. The first patients described outside Japan were of Spanish or US origin (Caucasian or Hispanic) and were reported as having CANDLE syndrome [76]. The two families diagnosed with JMP syndrome, lacking the inflammatory symptoms of CANDLE and NNS/JASL, were of Mexican and Portuguese origin [77]. In 2010–2011 several groups reported that PRAAS syndromes were all due to homozygous mutations affecting the Proteasome subunit beta type-8 (*PSMB8*) gene, that encodes for the β5i subunit of the proteasome [68–71]; β5i is one of the three catalytic subunits (together with β1i and β2i) that are isoforms constitutionally expressed in the hematopoietic lineages and induced in non-hematopoietic cells by inflammatory cytokines such as IFN-γ [78]. The proteasome variant containing the β1i, β2i, and β5i isoforms is called immunoproteasome. The *PSMB8* gene is expressed in two main transcripts of 272 aa (transcript ENST00000374881) or 276 aa (transcript ENST00000374882). All Japanese patients described carry the same missense mutation (variant ID rs387906680, referred as G197V or G201V depending on the transcript used as reference) [70, 71], while Mexican, Portuguese, Spanish, and Hispanic patients share the T75M mutation; a patient of Ashkenazi Jewish origin carried a C135X homozygous variant [69]. Interestingly, two patients (one from the US and one from Spain) who carried only a heterozygous T75M variant where subsequently found to have a further deleterious mutation in another subunit of the proteasome, *PSMA3* [79]. In the same publication, novel CANDLE-associated mutations were described in the previously unreported *PSMA3*, *PSMB4* and *PSMB9* proteasomal subunits and the proteasomal associated protein, POMP, in 5 patients of Jamaica, Irish and Palestinian origins. Importantly, through peripheral blood gene expression profiles and in vitro knock-down experiments in primary cells derived from affected patients, PRAAS were clearly associated to type I IFN induction.

Taken together, all these reports clearly link proteasome-related gene mutations to the type I IFN inflammatory response seen in PRAAS.

#### Spondyloenchondrodysplasia

Homozygous mutations of the tartrate-resistant acid phosphatase gene (*ACP5*), encoding for the protein TRAP, cause the immune-osseous disease, SPENCD [80, 81], which is characterized by platispondily, enchondromatosis, brain calcifications, spasticity and autoimmunity including SLE with malar rash, lupus nephritis, antiphospholipid syndrome and anti-dsDNA antibodies. The first case was originally described in a patient with juvenile SLE and bone abnormalities [82]. Patients present increased type I IFN signature in peripheral blood [80], serum, urine and dendritic cells accumulation of the TRAP substrate osteopontin (OPN), and Th1 polarizing cytokine production by dendritic cells (DC) [81]. Although the mechanism of type I IFN deregulation in SPENCD is not clear yet, it seems to be linked at least in part to an increased signalling through the TLRs, as it has been shown in mice that OPN is essential downstream of TLR9 for IFN-α production in plasmacytoid-DC [83].

## Other monogenic interferonopathies with less severe inflammatory phenotype

### Aicardi-goutieres syndrome and ISG15 deficiency

AGS is a progressive encephalopathy with neonatal (or possibly fetal) onset associated with an increase in white blood cells count and IFN-α concentration in the cerebrospinal fluid, basal ganglia calcifications in the absence of congenital infections. The presentation resembles that caused by transplacental-acquired infections and originally it was referred to as pseudo-TORCH (Toxoplasma, Rubella, Cytomegalovirus and Herpes simplex). A part from the severe neurological phenotype, over time patients develop glaucoma, chilblains and autoimmune features similar to typical SLE [84]. As suggested by Gresser and colleagues [17], type I IFN is thought to play a critical role in the disease pathogenesis and almost all patients present a strong IFN signature in peripheral blood [67]. The genes mutated in AGS are *TREX1* (AGS1, OMIM #225750), *SAMHD1* (AGS5, OMIM #612952), *RNaseH2A* (AGS4, OMIM #610333) *RNASEH2B* (AGS2, OMIM #610181), *RNASEH2C* (AGS3, OMIM #610329), *ADAR1* (AGS6, OMIM #615010), *IFIH1* (AGS7, OMIM #615846). A less severe phenotype has been described in patients presenting with idiopathic basal ganglia calcification (IBGC), seizures and autoantibodies, and harboring mutations in the *ISG15* gene (IMD38, OMIM #616126) [85].

### Singleton-merten syndrome

Singleton-Merten syndrome (OMIM #182250) is an AD disorder characterized by dental abnormalities (e.g. delayed primary tooth exfoliation, permanent tooth eruption and tooth loss, not present in the atypical form, OMIM #616298) aortal and hearth valve calcifications, skeletal abnormalities (distal limb osteolysis, widened medullary cavities), psoriasis, and glaucoma [86]. Affected patients carry a specific missense gain-of-function mutation in *IFIH1* or *DDX58* genes, dsRNA-receptors that activate type I IFN responses. Not surprisingly, both patients with Singleton-Merten and atypical Singleton-Merten syndrome present with increased type I IFN activity in peripheral blood [28, 29].

### Diagnostic approach

The diagnosis of type I interferonopathies can be elusive, especially for patients presenting mainly with flares of inflammatory symptoms without neurological or skeletal involvement. Atypical or incomplete SLE-like symptoms occurring in infancy or in preprepubertal age; sings of vasculopathy such as skin ulcers, chilblains and strokes; panniculitis with or without lipodystrophy, and interstitial lung disease in the context of systemic inflammation should always rise the suspect of a type I interferonopathy.

Early-onset necrotizing vasculitis, thrombotic vasculopathies and granulomatous polyangiitis cANCA-related have to be considered in the differential diagnosis. Moreover chronic bronchiolitis, immune deficiencies associated with follicular bronchiolitis and LIP, pulmonary hemorrhages due to collagen vascular diseases, and metabolic diseases such as prolidase deficiency and lysinuric protein intolerance should be ruled out.

Studies in AGS have demonstrated the strong correlation between mutations in AGS-related genes and type I interferon signature [67]. Using six ISGs derived by previous studies in SLE [87, 88], Rice et al. developed a score (named “interferon score”) with a high sensitivity for AGS. Detection of ISGs upregulation in peripheral blood has been used also in patients with other interferonopathies, in particular PRAAS [79], suggesting the potential relevance not only as a research biomarker, but also as a screening and diagnostic tool. Accordingly, we are currently assessing the efficiency of combining the interferon signature and targeted next generation sequencing for the diagnosis of type I interferonopathies in pediatric rheumatic undifferentiated patients (manuscript in preparation).

Definitive diagnosis for patients with clinical presentation suggestive of type I interferonopathy, positive interferon score and no mutations detected in known disease-related genes (Tables 1 and 2) can be attempted taking advantage of modern next generation sequencing approaches, such as whole exome or whole genome sequencing.

### Therapeutic options

Development of definitive therapeutic indications for type I interferonopathies has been extremely challenging due to the i) variability of clinical presentation even within the same genotype ii) rarity of the patients and only recent identification of most of the molecular causes iii) difficulty in assessing disease response, and iv) resistance to conventional therapies.

Commonly, patients are treated with high doses of intravenous methylprednisolone, oral prednisone and intravenous immunoglobulins during the acute phases with often only partial control of the flares. Disease-modifying antirheumatic drugs (DMARDS) such as methotrexate, mycophenolate-mofetil, antimalarians and azathioprine as well as biologics such as infliximab, etanercept, anakinra, tocilizumab, and rituximab have been anecdotally used and resulted ineffective in most cases [30, 31, 63–65, 89–91].

As explained above, type I interferon pathway represents the common pathogenic mechanism of these different diseases. In vitro experiments in patient-derived primary cells suggest that inhibition of this pathway is the most promising therapeutic strategy. Different drug targets have been identified and reviewed recently [92]. Particularly promising is the blockade of IFNAR signaling through JAK inhibitors. A clinical trial for the compassionate use of the drug Baricitinib, an oral JAK1/2 inhibitor under FDA approval consideration for rheumatoid arthritis and in phase 2 development for atopic dermatitis and diabetic nephropathy, is currently ongoing at the national institute of health (NIH) for patients with CANDLE, SAVI, and juvenile dermatomyositis (clinical trial identification number: NCT01724580) and has shown promising results [93]. Sporadic experience of compassionate use of Ruxolitinib, an oral JAK 1/2 inhibitor FDA approved for polycythemia vera and myelofibrosis and in phase 2 development for rheumatoid arthritis and alopecia areata, have also shown preliminary positive results ([94] and our center, manuscript in preparation). However follow-up data about the effectiveness and safety of these drugs are still lacking.

Monoclonal antibodies targeting IFN-α (Sifalimumab) and IFNAR (Anifrolumab) are also a very promising therapeutic option in all type I interferonopathies. Phase 2 trials for adult SLE have been concluded for both Sifalimumab (NCT00979654) and Anifrolumab (NCT01438489) and a phase 3 trial for Anifrolumab in SLE is recruiting subjects (NCT02547922). Results seem to be promising, even if preliminary [95–97].

Given the possible role of endogenous retroviruses in the activation of nucleic acid receptors in AGS, a phase 2 trial with reverse transcriptase inhibitors (NCT02363452) has been developed and is currently recruiting patients.

## Conclusions and future directions

The study of patients with rare genetic diseases has revealed a central role of abnormal nucleic acid recognition and type I IFN pathway activation in human diseases characterized by autoinflammation and autoimmunity. Patients with type I IFN diseases are difficult to diagnose and usually resistant to common therapies. Thanks to the rapid advancement of sequencing techniques and the awareness of the existence of these new type of diseases, we anticipate that a growing number of patients seen by pediatric rheumatologist will be diagnosed as suffering from known or new type I interferonopathies. On the other hand, as already observed in other inherited autoinflammatory diseases (i.e. cryopyrinopathies), the pathogenic insights deriving from the study of these ultra-rare disorders, might represent a crucial turning point also for a number of frequent multi-factorial inflammatory diseases, such as SLE. For both families and clinicians this will represent a long-sought medical answer and a renewed hope for the identification of efficacious therapeutic approaches.

### Clinical data

Clinical data and blood samples for the analysis of the interferon signature where collected with written parental consent approved by Istituto Gaslini review board. Patient’s parents agreed to the publication of the images in Fig. 2.

## Abbreviations

ACP5: acid phosphatase 5, tartrate resistant
ADAR1: adenosine deaminase acting on RNA 1
AGS: aicardi-Goutières syndrome
ANA: anti-nuclear antibody
ANCA: anti-cytoplasmic antibody
ATIL: anti-TNF induced lupus
CANDLE: chronic atypical neutrophilic dermatosis with lipodystrophy and elevated temperature syndrome
CAPS: cryopyrin-associated periodic syndrome
cGAMP: cyclic di-GMP-AMP
cGAS: cyclic GMP-AMP synthase
CHBL: chilblain lupus
DC: dendritic cells
DDX58: DEAD box protein 58
ER: endothelial reticulum
ERGIC: endothelial reticulum-Golgi intermediate compartment
FMF: familial Mediterranean fever
GAS: gamma-activated sequences
HUVS: hypocomplementemic urticarial vasculitis syndrome
IFI16: γ-interferon-inducible protein 16
IFIH1: IFN-induced helicase C domain-containing protein 1
IFN: interferon
IFNAR: interferon-α receptor
IRF: interferon regulatory factors
ISG15: interferon-stimulated gene 15
JAK: Janus kinase
JASL: Japanese autoinflammatory syndrome with lipodystrophy
JMP: joint contractures, muscle atrophy, microcytic anemia and panniculitis-induced lipodystrophy syndrome
MAVS: mitochondrial antiviral-signaling protein
MDA5: melanoma differentiation-associated protein 5
MSMD: Mendelian susceptibility for mycobacterial disease
NLR: NOD-like receptors
NNS: Nakajo-Nishimura syndrome
OMIM: on line Mendelian inheritance in men
OPN: osteopontin
PRAAS: proteasome associated autoinflammatory syndromes
PSMB8: proteasome subunit beta type-8
RCVL: retinal vasculopathy with cerebral leukodystrophy
RIG-I: retinoic acid-inducible gene 1
RLR: RIG-I-like receptors
RNASEH2: ribonuclease H2
SAIDs: systemic autoinflammatory diseases
SAMHD1: deoxynucleoside triphosphate triphosphohydrolase SAM domain and HD domain 1
SAVI: STING associated vasculopathy with onset in infancy
SLE: systemic lupus erythematosus
SMS: singleton-merten syndrome
SOCS1: suppressor of cytokine signaling 1
SPENCD: spondyloenchondrodysplasia
STING: stimulator of interferon genes
TBK1: TANK-binding kinase 1
THES: trichohepatoenteric syndrome
TLR: toll-like receptors
TMEM173: transmembrane protein 173
TNF: tumor necrosis factor
TRAP: tartrate-resistant acid phosphatase
TRAPS: tumor necrosis factor-associated periodic syndrome
TREX1: DNA 3ʹ _repair exonuclease 1
USP18: ubiquitin-specific protease 18
